# Periocular Data Fusion for Age and Gender Classification

**DOI:** 10.3390/jimaging8110307

**Published:** 2022-11-09

**Authors:** Carmen Bisogni, Lucia Cascone, Fabio Narducci

**Affiliations:** Department of Computer Science, University of Salerno, I-84084 Fisciano, SA, Italy

**Keywords:** periocular features, multimodal fusion, machine learning, fusion strategies, privacy

## Abstract

In recent years, the study of soft biometrics has gained increasing interest in the security and business sectors. These characteristics provide limited biometric information about the individual; hence, it is possible to increase performance by combining numerous data sources to overcome the accuracy limitations of a single trait. In this research, we provide a study on the fusion of periocular features taken from pupils, fixations, and blinks to achieve a demographic classification, i.e., by age and gender. A data fusion approach is implemented for this purpose. To build a trust evaluation of the selected biometric traits, we first employ a concatenation scheme for fusion at the feature level and, at the score level, transformation and classifier-based score fusion approaches (e.g., weighted sum, weighted product, Bayesian rule, etc.). Data fusion enables improved performance and the synthesis of acquired information, as well as its secure storage and protection of the multi-biometric system’s original biometric models. The combination of these soft biometrics characteristics combines flawlessly the need to protect individual privacy and to have a strong discriminatory element. The results are quite encouraging, with an age classification accuracy of 84.45% and a gender classification accuracy of 84.62%, respectively. The results obtained encourage the studies on periocular area to detect soft biometrics to be applied when the lower part of the face is not visible.

## 1. Introduction

Soft biometrics is an area of research which has gained much attention in the recent years. It allows the estimation of age, gender, race, etc., based on human face, hand veins, eyes, keystroke analysis, or any other physical and behavioral personal trait. A unimodal biometric system, that is based on a single biometric characteristic, has several problems and limitations due to the lack of data, a poor quality of the information collected or, as in the case of soft biometrics, a low discriminatory power. To overcome these issues, a multi-biometric system, i.e., a system that merges different biometric features, can help to improve performance and consolidate the information obtained. The fusion can take place considering different sources and at different levels. One of the questions that arises most is what to merge [1]. The system can be described as follows, depending on what it combines:

- Multi-sensor: these systems leverage multiple sensors to obtain information on a single biometric trait [2]. This strategy is particularly suitable when the sensors to grab the desired characteristic are all available and properly running.
- Multi-algorithm: the basic idea is being able to extract different characteristics from the same sample by applying different algorithms [3]. If the characteristics extracted from two different algorithms are complementary, the performance of the entire system can improve.
- Multi-instance: the advantages of this approach are lower sensitivity to noise due to the higher number of sample acquisition, greater similarities within a certain class and greater variability between them [4]. For example, in the case of iris recognition, both the left and right iris are used [5].
- Multi-sample: these systems use multiple samples of the same biometric trait, often they are captured with some variations. For example, if you want to create a facial recognition system, you can extract information from the same video, combining the data acquired from a single sensor on multiple video frames [6].
- Multi-modal: in this approach, multiple biometric traits are analyzed. It is possible to create a system that combines physical and/or behavioral characteristics. Such a choice may be more appropriate when security is crucial to protect sensitive data [7].

**Figure 1:**
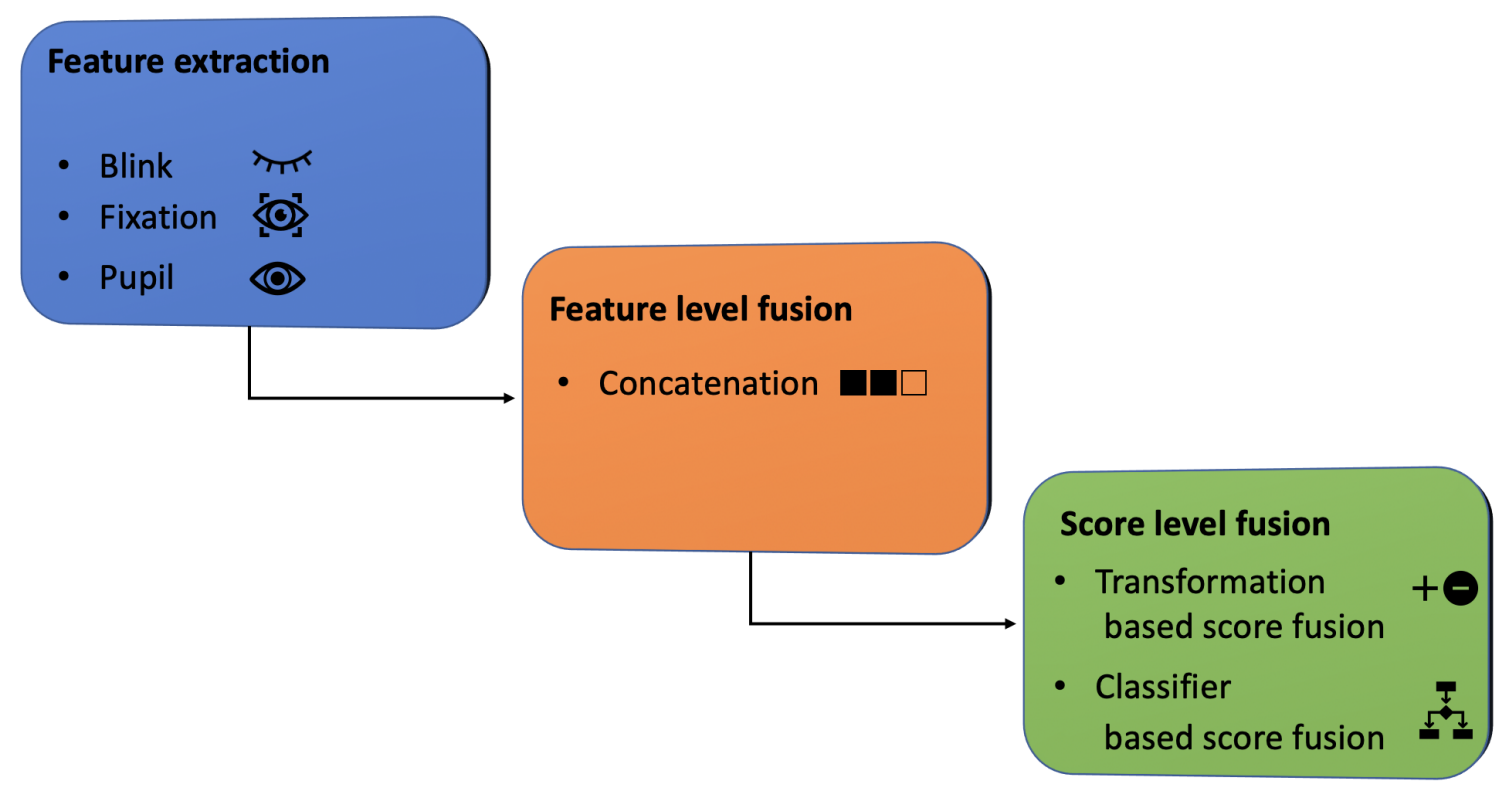
The workflow of the proposed fusion strategy.

Once the data has been acquired, depending on the type of information available, it is possible to define different levels of fusion. These strategies can be divided into two macro-areas: pre-classification with a fusion *before* the matching (sensor level, feature level) and post-classification, with a fusion *after* the matching (score level, rank level, decision level). Feature level fusion is achieved by combining the different feature vectors that are extracted separately from each biometric trait. Concatenation or summation [8] are examples of this class of approaches. Sensor-level fusion is usually associated with a strategy of multi-sensor or multi-algorithm, where raw information is combined immediately after its acquisition. A rank-level fusion is applied when a ranked list of matching identities can be obtained from each algorithm [9]. The decision level fusion combines the answers of the different algorithms to obtain the final decision. The main advantage is that it is particularly suitable when only the final decision is available [10]. Score level fusion is a post-classification process in which the scores produced by different algorithms are combined [11]. In this work, using only the information provided by the periocular area, we investigated how effectively fusion approaches that combine pupils, fixations, and blinks, can estimate the gender and age group of users. The estimate of belonging to one of the two classes can be particularly useful as information to be integrated or as the only confidence estimator. In the first case, for example, it can be integrated to improve the performance of iris recognition techniques, while, in the second case, it can be a good estimator in the presence of partial occlusions in challenging contexts (such as, for example, during a pandemic epidemic when it is mandatory to wear a mask when carrying out common daily activities). To do this, a data fusion strategy is implemented (Figure 1). First, we adopted a concatenation scheme for fusion at the feature level while, at the score level, we applied and then evaluated the performance of transformation and classifier-based score fusion methods. The main contributions of the work can be summarised as follows:

- According to our knowledge, this is the first paper to combine numerical data derived from the periocular area for the purposes of demographic recognition.
- Extensive testing of the optimal setup of classifiers for usage in a fusion scenario.
- An ad hoc experiment designed to evaluate the combination of the two investigated fusion techniques: feature-based and score-based fusion.

The rest of the paper is organized as follows: Section 2 provides a brief overview about the works in literature on periocular features. We discuss the theoretical aspect of transformation and classifier-based score fusion methods in Section 3. The experimental protocol, as well as the description of the dataset, is reported in Section 4. Section 5 presents a discussion and evaluation of the results obtained. Finally, the paper is concluded in Section 6.

## 2. Related Work

### 2.1. Periocular Features

The activity of classification by age and gender has acquired great importance in recent years due to the interest in profiling people on social media [12]; with the goal of advertising fields to customize the choice of customers [13], etc. The information for these two activities can be extracted from different physical and/or behavioral traits such as the face, 2D estimated skeletal points [14], gait [15], socio-linguistics features for a posted content on social media, and many others. Among the various biometric traits available, in this work, we focus on three features extracted from the periocular area: pupil size, duration, and number of blinks and fixations.

#### 2.1.1. Pupil

The literature presents several work aiming at discussing how to relate the variation in diameter of the pupil to specific causes. The reasons can be varied as physical factors, such as light; chemical factors, such as drug use; or cognitive and/or emotional factors. Pupil dilation has been used successfully to predict differences in stress levels. In [16], the authors studied the impact of acute stress on different emotion regulation strategies in men and women. The subjects participating in the experiment were asked to regulate their emotions in response to negative images using various techniques that had been previously explained to them, such as reappraisal or distraction. The effects of stress in relation to personal affective assessments and pupil expansion highlight interesting links between stress levels, sex, and emotion regulation, providing important information on gender differences. Stressed men exhibited notable pupil diameter measures during the reappraisal. Guillon et al. [17] highlighted age as one of the most significant factors that influences pupil size. In fact, also in this work, it was shown that smaller diameters are recorded in older groups and larger pupils in young people. The pupil size decreases significantly with increasing age. The pupil, therefore, lends itself to being both a physical and a behavioral biometric trait. The pupil, therefore, lends itself to being a biometric trait, both physical and behavioral, also useful for demographic classification, i.e., respect to age and gender [18]. Through an extensive study, Cascone et al. [19] analyze the size and dilation of the pupil over time with the aim of obtaining a classification of people by age and gender. The work suggests that promising results can be obtained in age classification, in contrast to gender recognition which is a more complex problem due to the influence of several different factors.

#### 2.1.2. Blink

Blinking is a self-regulating, natural, and instinctive response, that allows keeping your eyes healthy and clean. The various parameters associated with blinks, such as duration, frequency, speed, and latency, can be used to extract information on the response of subjects to different stimuli. From the existing works in the literature, it seems evident that the existence of a continuum between a very low blink rate, relative to those performances that require high visual attention, and the increase in blink frequency just before sleepiness and during boring tasks. Sakai et al. [20] exhibited that the blink rate decreases when visual attention is required, while increases when the state of visual attention switched from attentive to inattentive. This biometric trait is mainly used to investigate between moments of visual attention and fatigue, but little explored for demographic classification tasks. However, the few articles presented in this sense seem to give some interesting ideas. In [21], for example, the authors showed that women blink numerically more often than men (15.2 times vs. 13.3 times per minute).

#### 2.1.3. Fixation

The saccade is a rapid eye movement performed to shift a peripheral region to the center of the visual field. The time between two saccades is generally called fixation. This characteristic indicates how people acquire information. It happens in a range varing from a few milliseconds to several seconds. A high number of fixations can suggest difficulties in interpreting information while their longer duration can have different interpretations: more demanding cognitive processes, greater interest, etc. In [22], the authors investigate the use of different fixation measures as memory indicators during facial recognition lies. They recorded fewer fixations when subjects voluntarily denied recognition of familiar faces than when they correctly pointed to faces that were not actually known to them. Moss et al. [23] extracted different eye features when participants were shown natural images. They observed how women, on average, tended to be more exploratory, making more fixations for those images where there were no faces. Furthermore, for heterosexual couples, it was observed that all participants preferentially stared at female figures, with a higher percentage recorded in women (61% for female figures; 39% for male figures) than men (53% for figures female; 47% for male figures). The fixations of men tended to focus only on the face, unlike the female ones which were more varied. The fixations in relation to the age have been little explored.

### 2.2. Data Fusion

Multi-biometric systems merge information from multiple biometric sources. Typically, including more characteristics compensates for the inherent limitations of a single trait. The outcomes demonstrate superior performance, reliability, and robustness in comparison to uni-biometric systems. For these reasons, multi-biometrics is progressively becoming common in real-world applications [11]. Scoring-based techniques are undoubtedly among the most popular. There have been numerous score fusion techniques proposed in the literature. There are three basic categories of score combining rules: transformation, classification, and density fusion strategies. Combining the benefits of each group can result in a robust fusion process. The first two appear to be among the most prevalent in literary works. In [11], the authors propose a hierarchical combine network to integrate different fusion approaches from transformation- and classification-based categories into a single framework for classification. The involved modalities are periocular and iris. It is evident from the work that by implementing this approach it is possible to achieve a higher level of verification precision than with a single component-based approach. The same conclusions also true for the work [24]. In addition to utilizing a fusion technique at the score level, the authors test one at the level of features in this study. Two fusion strategies are therefore examined: feature level combining using the sum and concatenation methods, and score level merging using the max approach to blend selected deep features. The best result is achieved with a feature level fusion with sum. Rather than analyzing them separately, in [25] the authors deal with the problem of fusion by combining both levels of fusion (feature level and score level). In particular, the proposed fusion scheme is one in which three traits (fingerprint, palmprint, and earprint) are combined at two levels, feature level and score level. The performance of the individual matchers is analyzed to choose two of the three modalities that should then be used for feature level fusion. The idea behind the choice of these two traits is that they must be improved to the maximum in order to improve the recognition capacity through their fusion at the feature level. They combine the codes rather than the raw characteristics of these two modalities. The rationale behind using coded characteristic values is to reduce processing time and extract useful information from each of the two selected biometrics. Next, they apply a score level strategy.

## 3. Methodology

### 3.1. Transformation-Based Score Fusion

Transformation-based score fusion is the most intuitive and used score level fusion technique, as it is simple to make. For a given sample, it allows to combine the scores obtained from the different algorithms $\left(s_{1},s_{2},\cdots ,s_{n}\right)$ and generate a new unique score (S), using a function (f), to which these previously generated normalized or standardized scores are given as input. So, it consists of simple algebraic manipulation of the scores through a specific function showed in Equation (1).

(1) $$S=f(s_{1},\cdots ,s_{n})$$

The transformation-based fusion techniques used in this work are weighted sum, weighted product, and the Bayesian rule.

#### 3.1.1. Weighted Sum

The weighted sum is a variant of the arithmetic sum. Unlike the arithmetic sum, here the score vectors are first multiplied by a weight $w_{i}$ and then added together. Let $\left(s_{1},\cdots ,s_{n}\right)$ be the score vectors obtained by the *n* algorithms. The formula for obtaining the weighted sum is presented in Equation (2).

(2) $$S=\sum_{i=1}^{n}w_{i}s_{i}$$

with $0\leq w_{i}\leq 1$. The weight is directly proportional to the importance of the associated score.

#### 3.1.2. Weighted Product

The weighted product is obtained from a variant of the classic arithmetic product. Let $\left(s_{1},\cdots ,s_{n}\right)$ score vectors obtained from *n* algorithms, its formula is presented in Equation (3).

(3) $$S=\prod_{i=1}^{n}s_{i}^{w_{i}}$$

where $w_{i}$ is the weight of the algorithm *i*. It can be observed how, in this case, the scores influence each other more than the weighted sum. For example, if one of the scores is close to 0, the score obtained from the fusion will also be close to 0.

#### 3.1.3. Bayes Fusion Rule

Bayes’ rule is one of the fundamental pillars in probabilistic theory. The definition of this rule for the event *x* and *y* is defined in Equation (4).

(4) $$p\left(x|y\right)=\frac{p(y|x)p(x)}{p(y)}$$

If we have a score matrix M and different classes *i*={1,2,⋯} to which our observations can belong, the Bayes’ rule can be rewritten as in [26] resulting in Equation (5).

(5) $$p\left(i|M\right)=\frac{p(M|i)p(i)}{p(M)}$$

where p(i) is marginal probability of *i*, p(M|i) is the conditional probability, $p\left(M\right)=\sum_{i=1}^{n}p\left(M|i\right)p\left(i\right)$ is the evidence and *n* is the number of classes. Assuming that the scores are conditionally independent given the classes, p(M|i) can be rewritten as in Equation (6).

(6) $$p\left(M|i\right)=\prod_{j=1}^{n}p_{j}\left(s_{j}|i\right)$$

where $p_{j}\left(s_{j}|i\right)$ is the score of *j*-th algorithm related to the *i*-th class. So, let $t_{a}$ and $t_{b}$ the scores obtained by two algorithms for a binary classification problem, their fusion through the Bayes function is given by [27,28] as in Equation (7).

(7) $$S=\frac{t_{a}\times t_{b}}{\left(1-t_{a}\right)\left(1-t_{b}\right)+\left(t_{a}\times t_{b}\right)}$$

Additionally, in this case, to give greater importance to one biometric over another, we have assigned a weight $\left(w_{i}\right)$, to be multiplied to the vector of the score which must have a lower value for the final decision.This weight tends to reflect the accuracy achieved by the algorithms involved. If one algorithm tends to have a better performance than another, the prediction of the first should have a greater weight when making a choice.

### 3.2. Classifier-Based Score Fusion

In classifier-based score fusion, score vectors obtained by biometric algorithms are considered as feature vectors that are, in turn, discriminated as genuine or impostor scores. Therefore, the classifiers, learn the relationship between the various score vectors, treated as the new characteristics, which are used to solve the classification task. On the basis of this new dataset, the selected algorithm learns a decision boundary between the two classes. In this work, different classifiers were used to consolidate and improve the scores obtained to arrive at a final decision. The classifiers are first trained with the labeled score data and then tested with unlabeled ones. In this work, we mainly focused on four groups of ML algorithms: Decision Tree, ensemble, optimization, and instance-based algorithms. Decision tree (DT) methods are based on a tree structure where the inner nodes represent the features, the leaves represent the outcomes, and the branches represent the decision rules [29]. The goal of the ensemble methods is, instead, to combine the responses of several learning estimators in order to improve the predictive performance and robustness of a single algorithm. These kind of methods can be divided in two families: averaging and boosting methods. The former are Random Forests (RF) [30] and Bagging (BG) classifiers [31]. The basic idea is to independently calculate the average of the predictions of different estimators. This ensures that the variance of a single base estimator is reduced, The second group of methods includes AdaBoost (AD) [18] and Gradient Boosting (GB) classifiers [32]. The driving principle is to have a strong classifier from the combination of weak ones. The base estimators are used sequentially to correct the errors from the previous model. Instance-based learning strategy is a decision-making problem based on instances seen in the training phase that are deemed important or representative of the problem. These algorithms, called winner-takes-all methods, generate a database of sample data stored in memory and compare the new data for which you want to obtain a prediction through a similarity measure in order to find the best match. Among the most famous and used methods belonging to this class appear K-Nearest Neighbor (KNN) [33] and Support Vector Machines (SVM) [34]. Stochastic Gradient Descent Classifier (SGD) [35] is an optimization algorithm that implements a simple learning routine of the descent of the stochastic gradient.

## 4. Experimental Protocol

### 4.1. Dataset

**Figure 2:**
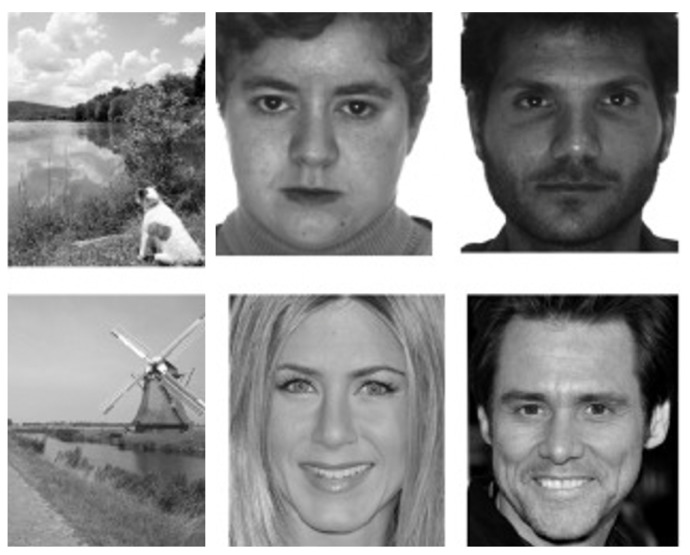
Some examples of images shown to participants during the data acquisition process in the GANT [36]. The first column shows images of two landscapes. The last two columns, on the other hand, show images of women and men: in the first row there are images of unknown people while in the second row there are images of two famous actors.

The experimental analysis was conducted on the GANT dataset [36]. A total of 112 volunteer participants (73 males and 39 females) took part in the experiment, divided as follows by age groups: 17–18 (11 subjects), 21–30 (58 subjects), 31–40 (9 subjects), 41–50 (16 subjects), 51–60 (8 subjects), 61–70 (9 subjects), and 71–80 (1 subject). All subjects had normal vision. Acquisitions were made using a Tobii 1750 remote eye tracker (1280 × 1024 screen resolution, 50 Hz sampling frequency). A total of 18 black-and-white pictures were randomly shown, 16 depicting human faces and 2 landscapes (examples in the Figure 2). The images of the faces are divided as follows: 8 of men, 8 of women, and for each division 4 are of famous people (such as actors) and the other 4 are of people unknown to the participants. All images have the same gray level distribution.

### 4.2. Data Pre-Processing

The eye tracker associates the validity codes (0–4) to each acquisition related to both eyes which gives a measure of how certain the instrument is to have found a specific eye. When the associated code is a low value it means that the instrument gives a high reliability value to the acquisition obtained. The first filtering operation consisted in the elimination of those acquisitions with respect to which one eye had been associated with a code other than 0. The other codes (1, 2, 3) can in fact refer to corrupt, incorrect acquisitions or in relation to which a single eye has been acquired by making assumptions whether it is the right or left one or without being sure which of the two is. Therefore, only those acquisitions were saved for which the instrument has associated code 0 or 4 for both eyes. In fact, a pair of gaze data with validity code 4 on both eyes, followed by a number of gaze data with validity code 0 on both eyes, is usually a sure sign of a blink. For each participant, data samples related to viewing each of the 18 images presented as stimuli were analyzed, synthesized, and organized through different statistical indicators including indices of dispersion and position. For each image, the characteristics extracted from the pupil were studied in terms of their diameter measurement while those relating to fixations and blinks in terms of duration and number. Specifically, for blinks, a counter of “fast” blinks is also increased, i.e., those for which it is not possible to actually calculate the duration. A total of 3665 values are obtained. Since the samples obtained were gender-unbalanced, a randomly selected subset was analyzed.

**Figure 3:**
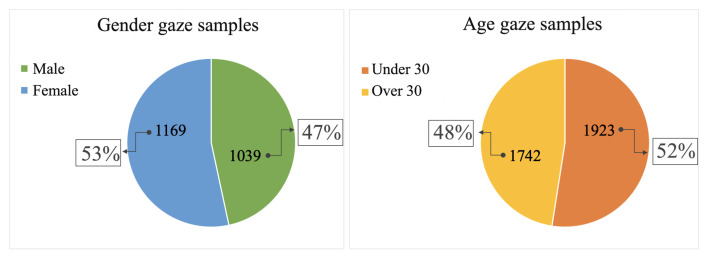
Distribution of data samples with respect to the two classification tasks.

More details on the number of samples analyzed per classification task are given in the Figure 3. Based on the number of participants per age group (as discussed in the Section 4.1) it was decided to divide the subjects into two groups, those under the age of 30 and those over (Figure 3).

### 4.3. Fusion Strategies Implementation

Feature vectors obtained as explained in Section 4.2 are combined through the feature level fusion by a concatenation strategy. This strategy was preferred to a summation one that can reduce dimensionality because in this case, compared to the three biometric modalities (pupil, fixation, blink) we have no redundant information. In fact, applying the Spearman non-parametric test between the characteristics of the three modalities pairs (blink–pupil, fixation–pupil, blink–fixation), we observed that there is poor correlation between them. For example, studied the pupil–blink pair, we applied this test to all possible pairs of characteristics that could be obtained by comparing these two biometric traits. We report in Table 1 the average of these values for each pair analyzed.

**Figure 4:**
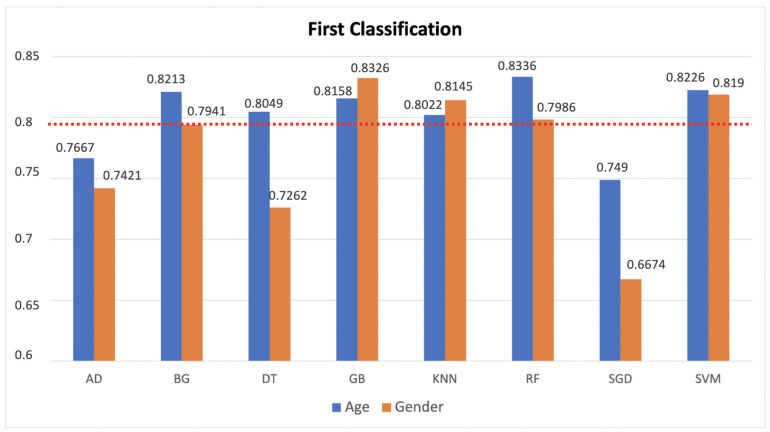
The results of the age and gender classification using different classifier. The accuracies over the red line are taken in considerations.

So, it is evident how the association between these modalities is very poor. Then, the feature vectors were transformed by scaling each feature over a given interval. The rationale behind this choice is that this scaling includes robustness to very small standard deviations of features and the retention of zero entries in the sparse data. After these considerations and operations, the experimentation was conducted by partitioning the available data into a random train and test subsets with a ratio of 80:20, respectively. By dividing the entire dataset into these pairs of sets, we drastically reduce the number of samples that can be used for the learning of our choice models (see Section 3.2), and also we strongly tie the results obtained to the random selection of these subsets. Another problem is related to the chosen estimators, setting fixed parameters has an impact on the final performance of the system. To overcome these problems we used an exhaustive search on different parameter values specified for each estimator with a k-fold cross-validation procedure with a variable “k” between 2 and 10. The basic idea behind this procedure is to divide the training dataset into k parts and train the model on a subset consisting only of k − 1. The resulting part is used to validate the model with a performance measure such as accuracy. The measure of the performance obtained is the average of the k − 1 values derived individually. For each model trained, a confidence score associated with each element of the test set was generated with respect to each label (male/female or under30/over30), i.e., the probability that that particular subject in the test belongs to a category rather than to another. After obtaining these scores from the estimators, we used several strategies to combine them. We focused on two approaches: transformation-based score fusion and classifier-based score fusion. For the first strategy, only the scores of those classifiers were selected that reported an accuracy greater than a threshold (over the red line in Figure 4).

The techniques described in Section 3.1.1 and Section 3.1.2 were applied to the scores of the selected classifiers by matching them in all possible combinations. The best weights were calculated with brute force combinations of weights of the type w/10 with *w* ranging from 1 to 10.

**Figure 5:**
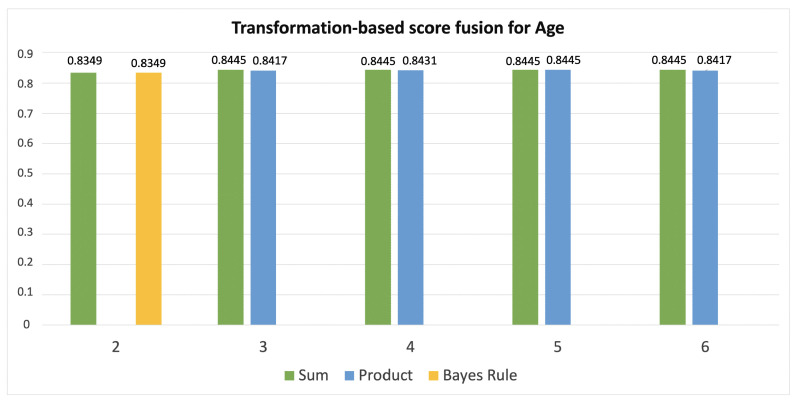
The results of combining 2, 3, 4, 5, and 6 classifiers in age recognition using sum, product and Bayes rule as fusion strategies.

**Figure 6:**
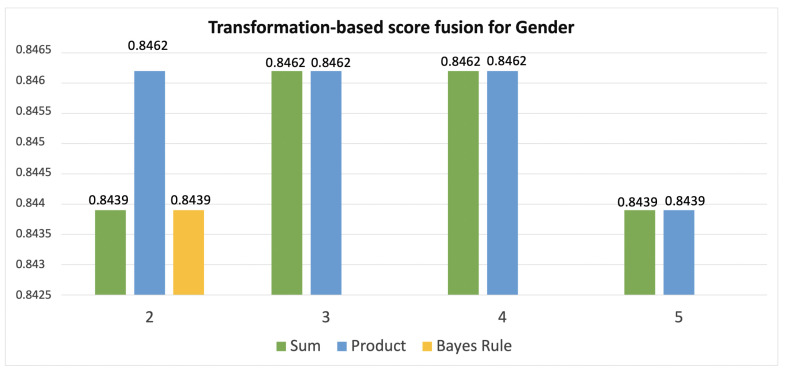
The results of combining 2, 3, 4, and 5 classifiers in gender recognition using sum, product and Bayes rule as fusion strategies.

Table 2 and Table 3 report the combinations of classifiers that have reported the highest score with respect to the number of models that are combined, therefore from 2 to 6 by age classification (Figure 5) and from 2 to 5 by gender one (Figure 6).

**Figure 7:**
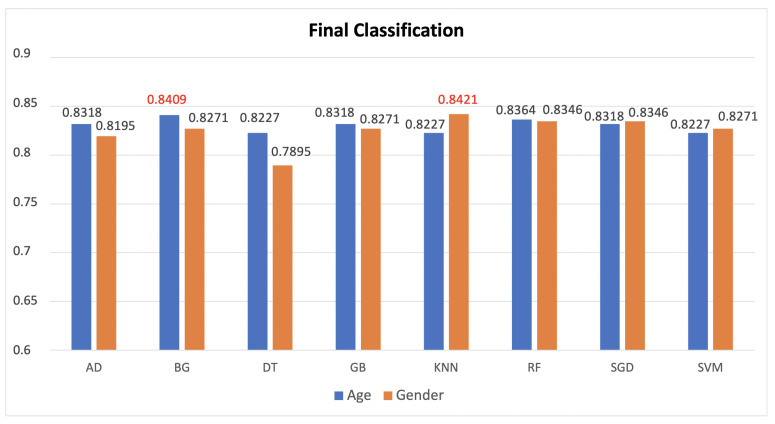
The results of the classifiers are obtained using the combination of the best two scores of SVM and Random Forest for age classification, and of Gradient Boosting and SVM for gender one. In red are reported the best accuracies for age and gender, respectively.

The highest accuracy for the combination was reported only when it actually exceeded the maximum accuracy reported in Figure 4 for each classification task. Another strategy, based on Bayesian rules, as explained in Section 3.1.3, was also applied for the transformation-based score fusion, combining the selected classifiers two by two. The best results are reported in Table 2 and Table 3. The second score level fusion technique implemented is the classifier based score fusion approach. The models chosen are the same ones used to obtain the scores. These classifiers were trained on a new train dataset obtained by concatenating the scores relating to the two classifiers that reported the highest accuracy in Figure 4. So, for age classification, we concatenated the scores of SVM and Random Forest, while for gender classification Gradient Boosting and SVM ones. The rate train:test chosen was 70:30. Additionally, in this case, an exhaustive research of the best parameters was applied and different k-fold strategies for the models were tested in order to obtain the best results reported in Figure 7.

## 5. Discussion

In this work, we applied different fusion strategies to periocular data samples (Figure 1) with the aim of obtaining a gender and age classification. After the extraction of the characteristics (as explained in Section 4.2) relating to each biometric trait considered, at first, we proceeded with a fusion at feature level, opting for a concatenation strategy. As reported in Table 1, it was observed that the characteristics under analysis for each biometric trait were highly independent to each other. To support the need for this choice to obtain more competitive performances, we have replicated the experiment without this prior step. The same exhaustive research was applied to the classifiers described in Section 3.2.

**Figure 8:**
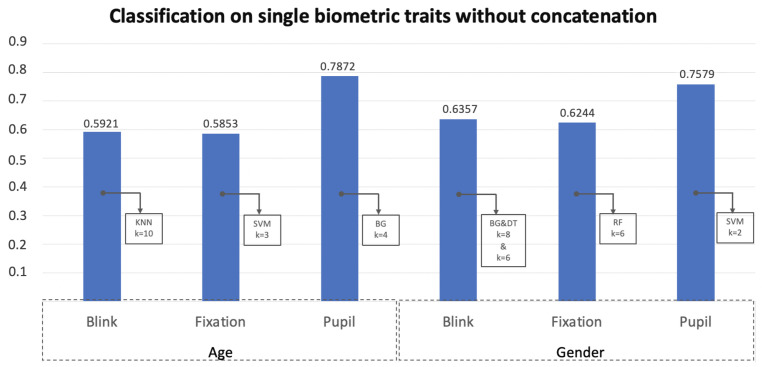
The results of the best classifiers on single biometric traits. k is the value of the implemented k-fold cross-validation strategy relating to the best accuracy achieved. Exploiting only the blink for gender classification there are two classifiers that report the same higher accuracy.

Figure 8 reports the accuracy achieved in the best configuration per biometric trait analyzed individually.

From this table, it is clear that of the three traits analyzed the pupil size is the most discriminating for both classification tasks. The blink and the fixations seem to perform poorer for the age classification showing comparable level of accuracy. For the gender classification, relating to blink, there are two classifiers (DT and BG) with which the same result is obtained. For this reason, the successive experiments were replicated using the scores of one and then those of the other. The combination of the scores of the classifiers through both the transformation and classification techniques are shown in the Table 4, Table 5, Table 6 and Table 7. By comparing the results of these tables with the final summary one (Table 8), it is possible to observe how, with the contribution of the concatenation, there is an increase in accuracy of almost 4% for the age classification and slightly higher for the gender. This is due to the initial accuracy which is significantly more unbalanced between the three biometric traits for the task of age classification. In any case, for gender it is necessary to take into account that in the score fusion the results shown in Figure 7 are related to the merger of the scores of the first two classifiers that gave the best results. In this case, in contrast, the best result (Table 5) is obtained by concatenating the scores of the three classifiers associated with the pupil, blinks, and fixations, respectively. Therefore, with the same accuracy reached, 84.21%, in the treatment without concatenation it is necessary to consider a greater number of classifiers to still have the same performance. Table 8 is a summary table of the results achieved. The best performances for both classification tasks are obtained in correspondence with the fusion strategy based on the transformation of the scores with respect to both the weighted sum and product, however, varying the number of algorithms involved. For the gender classification, to obtain the best performance, it is sufficient to consider only 2 classifiers and rely on the weighted product. Comparing the performances achieved with those in a previous work [19] it is evident that, only considering the pupil size higher performances are obtained by analyzing a smaller number of classifiers and no neural network models, e.g., multi-layer perceptron (useful pointing out that other statistical indices in addition to the average have bee used.) Through the proposed fusion strategy, feature level concatenation followed by transformation-based score fusion, the state of the art for gender classification using periocular features is exceeded by more than 25%. For the age classification, on the other hand, there is a slight increase. Although the maximum accuracy is reached with a neural network, if we refer to Table 5 of the paper [8] it is very evident that higher performances are obtained considering only the average and using the same classifier. Further studies may investigate which statistical indices are most appropriate and discriminatory for each task. However, a weighted sum of just three classifiers results in higher accuracy.

## 6. Conclusions and Future Developments

Data fusion is a useful solution to improve the performances and the synthesis of collected information, to be stored efficiently for frameworks operating in several different contexts. In biometric researches, the choice of working with soft biometrics is motivated by their high acceptability by acquired subjects. Combining the biometric traits the benefit is twofold: evaluate and store relevant soft features and preserve the privacy of sensitive data. The periocular area allows the extraction and analysis of numerous biometric characteristics. In various situations, such as the current COVID-19 pandemic, the restrictions that imposes the use of facial masks led various widely used biometric traits becoming ineffective due to possible occlusions (the facial masks for face recognition) or the need to have contact with the recognition system (practice currently not recommended to avoid further spread of the virus). For all these reasons biometric traits that do not need physical contact can be regarded an optimal choice for the recognition systems. In this work we use the information extracted from the variation of the pupil diameter, the duration and the number of blinks and fixations to focus on the demographic classification, i.e., consisting in age and gender recognition. These characteristics have been fused through different strategies. The results of our experiments show how effectively implementing a fusion strategy increases the performance of the entire system. A transformation-based score fusion appears to be preferred over a classification-based one. Furthermore, it is evident that the fusion of these characteristics has led to a significant improvement in terms of classification compared to the values obtained from the individual analysis. For gender and age, the best accuracy (84.62% and 84.45%, respectively) has been obtained both through a sum or a weighted product. It can be observed from the collected results that concatenation contributes to an increase in classification accuracy of approximately 4% for age and somewhat more for gender. The fusion technique based on the transformation of scores with respect to both weighted sum and product achieves the greatest performance for both classification tasks while adjusting the number of algorithms involved. To acquire the best performance in gender classification, it is sufficient to examine only two classifiers and rely on the weighted product. For gender classification, the proposed fusion technique of feature-level concatenation followed by score-based fusion yields performance that exceeds the state of the art by more than 25%. In terms of age classification, there is a slight increase. We can conclude that, although the periocular area has so far been little studied to derive a demographic classification, it could instead be an interesting biometric trait. In future investigations, we will further explore the potential of other biometric features related to the periocular area, such as saccades, as well as integrating information on pupillary variation in relation to time. Furthermore, our goal is to replace the brute force strategy for weight research with an approach based on genetic algorithms to solve the optimization problem of maximizing the accuracy achieved. Data fusion strategies can be useful for trust evaluation in challenging contexts such as, for example, during a pandemic outbreak when it is mandatory to wear a mask while performing common daily activities. The classification percentages obtained by machine learning algorithms can be used as a measure of trust of the estimated identity.

## Figures and Tables

**Table 1:** Spearman’s correlation coefficients with respect to the pairs of the three modalities.

	Spearman’s Correlation Coefficients
Blink-pupil	0.1439
Blink-fixation	−0.0992
Fixation-pupil	0.0644

**Table 2:** The results of the combination of the classifiers in gender recognition using different fusion strategies. *n* is the number of classifiers involved in the fusion process. The numbers in bold are the best results.

Transformation-Based Score Fusion for Gender			
	***n***	**Combination of Classifiers**	**Acc.**
Sum	2	GB&SVM SVM&KNN	0.8439
	3	GB&SVM&RF GB&SVM&BG	**0.8462**
	4	GB&SVM&RF&BG	**0.8462**
	5	GB&SVM&RF&BG&KNN	0.8439
Prod	2	GB&SVM	**0.8462**
	3	GB&SVM&BG	**0.8462**
	4	GB&SVM&BG&RF	**0.8462**
	5	GB&SVM&RF&BG&KNN	0.8439
Bayes Rule	2	GB&SVM SVM&KNN	0.8439

**Table 3:** The results of the combination of the classifiers in age recognition using different fusion strategies. *n* is the number of classifiers involved in the fusion process. The numbers in bold are the best results.

Transformation-Based Score Fusion for Age			
	***n***	**Combination of Classifiers**	**Acc.**
Sum	2	RF&SVM GB&SVM	0.8349
	3	GB&SVM&DT	**0.8445**
	4	SVM&KNN&DT&RF	**0.8445**
	5	GB&SVM&DT&KNN&RF GB&SVM&DT&KNN&BG	**0.8445**
	6	GB&KNN&DT&BG&SVM&RF	**0.8445**
Prod	2	-	**-**
	3	RF&SVM&DT GB&SVM&DT SVM&DT&BG	0.8417
	4	GB&SVM&KNN&DT SVM&KNN&DT&RF SVM&BG&DT&RF	0.8431
	5	SVM&BG&DT&RF&KNN	**0.8445**
	6	GB&SVM&BG&DT&RF&KNN	0.8417
Bayes Rule	2	RF&SVM	0.8349

**Table 4:** The results of the age classification are obtained from combination of the best scores of the three biometric traits. k is the value of the implemented k-fold cross-validation strategy relating to the best accuracy achieved. The numbers in bold are the best results. X indicates the features selected for experimentation.

Age Classification without Concatenation					
**Fixation**	**Pupil**	**Blink**	**Classifiers**	**k**	**Acc.**
X	X	X	KNN	2	0.8
	X	X	KNN	5	**0.8091**
X		X	AD	3	0.6182
	X	X	DT	2	0.7818
			BG	10	
			SVM	3	

**Table 5:** The results of the gender classification are obtained from combination of the best scores of the three biometric traits. For blinks, as maximum accuracy is achieved with two different classifiers, both scores are taken into account. For “Blink_1” we refer to the scores related to the DT classifier, while for “Blink_2” to those related to the BG classifier. k is the value of the implemented k-fold cross-validation strategy relating to the best accuracy achieved. The numbers in bold are the best results. X indicates the features selected for experimentation.

Gender Classification without Concatenation						
**Fixation**	**Pupil**	**Blink_1**	**Blink_2**	**Classifiers**	**k**	**Acc.**
X	X	X		SGD	4	0.8346
X	X		X	SVM	3	**0.8421**
X		X		KNN	6	0.7894
X			X	KNN	9	0.7669
	X	X		SVM	3	0.7970
	X		X	SVM	2	0.8045
				KNN	3	
				AD	2	
X	X			KNN	3	0.7970

**Table 6:** The results of the combination of the classifiers in gender recognition using different transformation-based score techniques without a a preliminary feature level fusion. The numbers in bold are the best results.

Transformation-Based Score Fusion for Gender without Concatenation				
	**Combination of Classifiers**			**Acc.**
	Fixation	Pupil	Blink	
Sum	RF	SVM	BG	0.8054
	RF	SVM	DT	**0.8167**
Prod	RF	SVM	BG	0.7443
	RF	SVM	DT	0.7511
Bayes Rule	RF	SVM		0.7579
	RF		BG	0.6923
	RF		DT	0.7059
		SVM	BG	0.7624
		SVM	DT	0.7851

**Table 7:** The results of the combination of the classifiers in age recognition using different transformation-based score techniques without a a preliminary feature level fusion. The numbers in bold are the best results.

Transformation-Based Score Fusion for Age without Concatenation				
	**Combination of Classifiers**			**Acc.**
	Fixation	Pupil	Blink	
Sum	SVM	BG	KNN	**0.7913**
Prod	SVM	BG	KNN	0.7763
Bayes Rule	SVM	BG		0.7804
	SVM		KNN	0.5921
		BG	KNN	0.7844

**Table 8:** For both classification tasks, the results of the single biometric traits (blink, fixation, and pupil) obtained with the same protocol are reported in the first three lines. The next line shows the best results obtained with our fusion strategy. In the last line there is a comparison with a paper that uses the same dataset with the same purpose. The numbers in bold are the best results.

Summary Table: Best Results							
	**Strategy**			**Features**	**Classifiers**	**k**	**Acc.**
**Age Classification**	First classification			Blink	KNN	10	0.5921
				Fixation	SVM	3	0.5853
				Pupil	BG	4	0.7872
				All	RF	6	0.8336
	Fusion	Sum	with conc.	All	GB&SVM&DT SVM&KNN&DT&RF GB&SVM&DT&KNN&BG GB&SVM&DT&KNN&RF	-	**0.8445**
			without conc.	Fixation Pupil Blink	SVM&BG&KNN	-	0.7913
		Prod.	with conc.	All	SVM&DT&KNN&BG&RF	-	**0.8445**
			without conc.	Fixation Pupil Blink	SVM&BG&KNN		0.7763
		Bayes	with conc.	All	RF&SVM	-	0.8349
			without conc.	Pupil Blink	BG&KNN	-	0.7844
		Classifiers	with conc.	All	BG	7	0.8409
			without conc.	Pupil Blink	KNN	5	0.8091
	[8]			Pupil	Multilayer perceptron	-	0.8369
**Gender Classification**	First classification			Blink	BG DT	8 6	0.6357
				Fixation	RF	6	0.6244
				Pupil	SVM	2	0.7579
				All	GB	2	0.8326
	Fusion	Sum	with conc.	All	GB&SVM&BG GB&SVM&RF GB&SVM&RF&BG	-	**0.8462**
			without conc.	Fixation Pupil Blink	RF&SVM&DT	-	0.81674
		Prod.	with conc.	All	GB&SVM GB&SVM&BG GB&SVM&RF&BG	-	**0.8462**
			without conc.	Fixation Pupil Blink	RF&SVM&DT	-	0.7511
		Bayes	with conc.	All	GB&SVM SVM&KNN	-	0.8439
			without conc.	Pupil Blink	SVM&DT	-	0.7851
		Classifiers	with conc.	All	KNN	4	0.8421
			without conc.	Fixation Pupil Blink	SVM	3	0.8421
	[8]			Pupil	SGD	-	0.5848

## Data Availability

Please contact the authors of [36].

## References

[1] Singh M., Singh R., Ross A. (2019). A comprehensive overview of biometric fusion. Inf. Fusion.

[2] Llano E., García Vázquez M., Vargas J., Fuentes L., Ramírez Acosta A. (2018). Optimized robust multi-sensor scheme for simultaneous video and image iris recognition. Pattern Recognit. Lett..

[3] Gomez-Barrero M., Galbally J., Morales A., Fierrez J. (2017). Privacy-Preserving Comparison of Variable-Length Data with Application to Biometric Template Protection. IEEE Access.

[4] Sudhakar T., Gavrilova M. Multi-instance Cancelable Biometric System using Convolutional Neural Network. Proceedings of the 2019 International Conference on Cyberworlds (CW).

[5] Rathgeb C., Busch C. (2014). Cancelable Multi-Biometrics: Mixing Iris-Codes based on Adaptive Bloom Filters. Comput. Secur..

[6] Goswami G., Vatsa M., Singh R. (2017). Face Verification via Learned Representation on Feature-Rich Video Frames. IEEE Trans. Inf. Forensics Secur..

[7] Jamdar S., Golhar Y. Implementation of unimodal to multimodal biometrie feature level fusion of combining face iris and ear in multi-modal biometric system. Proceedings of the 2017 International Conference on Trends in Electronics and Informatics (ICEI).

[8] Bokade G., Kanphade R. Secure Multimodal Biometric Authentication Using Face, Palmprint and Ear: A Feature Level Fusion Approach. Proceedings of the 2019 10th International Conference on Computing, Communication and Networking Technologies (ICCCNT).

[9] Sing J., Dey A., Ghosh M. (2019). Confidence factor weighted Gaussian function induced parallel fuzzy rank-level fusion for inference and its application to face recognition. Inf. Fusion.

[10] Kumar A., Kumar A. (2016). Adaptive management of multimodal biometrics fusion using ant colony optimization. Inf. Fusion.

[11] Algashaam F., Nguyen K., Banks J., Chandran V., Do T.A., Alkanhal M. (2021). Hierarchical fusion network for periocular and iris by neural network approximation and sparse autoencoder. Mach. Vis. Appl..

[12] Goshvarpour A., Goshvarpour A. (2019). Gender and age classification using a new Poincare section-based feature set of ECG. Signal Image Video Process..

[13] Htet K.S., Myint Sein M. Effective Marketing Analysis on Gender and Age Classification with Hyperparameter Tuning. Proceedings of the 2020 IEEE 2nd Global Conference on Life Sciences and Technologies (LifeTech).

[14] Barra P., Bisogni C., Nappi M., Freire-Obregón D., Castrillón-Santana M. Gender classification on 2D human skeleton. Proceedings of the 2019 3rd International Conference on Bio-Engineering for Smart Technologies (BioSMART).

[15] Barra P., Bisogni C., Nappi M., Freire-Obregón D., Castrillón-Santana M. (2019). Gait Analysis for Gender Classification in Forensics. Commun. Comput. Inf. Sci..

[16] Langer K., Hagedorn B., Stock L.M., Otto T., Wolf O., Jentsch V. (2020). Acute stress improves the effectivity of cognitive emotion regulation in men. Sci. Rep..

[17] Guillon M., Dumbleton K., Theodoratos P., Gobbe M., Wooley C., Moody K. (2016). The effects of age, refractive status, and luminance on pupil size. Optom. Vis. Sci..

[18] Cantoni V., Cascone L., Nappi M., Porta M. (2020). Demographic classification through pupil analysis. Image Vis. Comput..

[19] Cascone L., Medaglia C., Nappi M., Narducci F. (2020). Pupil size as a soft biometrics for age and gender classification. Pattern Recognit. Lett..

[20] Sakai T., Tamaki H., Ota Y., Egusa R., Inagaki S., Kusunoki F., Sugimoto M., Mizoguchi H. (2017). EDA-based estimation of visual attention by observation of eye blink frequency. Int. J. Smart Sens. Intell. Syst..

[21] Peddireddy A., Wang K., Svensson P., Arendt-Nielsen L. (2006). Influence of age and gender on the jaw-stretch and blink reflexes. Exp. Brain Res..

[22] Millen A.E., Hope L., Hillstrom A.P., Vrij A. (2017). Tracking the truth: The effect of face familiarity on eye fixations during deception. Q. J. Exp. Psychol..

[23] Moss F.J.M., Baddeley R., Canagarajah N. (2012). Eye movements to natural images as a function of sex and personality. PLoS ONE.

[24] El-Rahiem B.A., El-Samie F.E.A., Amin M. (2022). Multimodal biometric authentication based on deep fusion of electrocardiogram (ECG) and finger vein. Multimed. Syst..

[25] Kabir W., Ahmad M.O., Swamy M. (2019). A multi-biometric system based on feature and score level fusions. IEEE Access.

[26] Huber M.F., Merentitis A., Heremans R., Niessen M., Debes C., Frangiadakis N. Bayesian Score Level Fusion for Facial Recognition. Proceedings of the 2016 IEEE International Conference on Multisensor Fusion and Integration for Intelligent Systems (MFI).

[27] Akhtar Z., Fumera G., Marcialis G.L., Roli F. Evaluation of multimodal biometric score fusion rules under spoof attacks. Proceedings of the 2012 5th IAPR International Conference on Biometrics (ICB).

[28] Kim W., Song J., Park K. (2018). Multimodal biometric recognition based on convolutional neural network by the fusion of finger-vein and finger shape using near-infrared (NIR) camera sensor. Sensors.

[29] Swain P.H., Hauska H. (1977). Decision tree classifier: Design and potential. IEEE Trans. Geosci. Electron..

[30] Breiman L. (2001). Random forests. Mach. Learn..

[31] Dietterich T. (2000). Ensemble methods in machine learning. Lecture Notes in Computer Science (Including Subseries Lecture Notes in Artificial Intelligence and Lecture Notes in Bioinformatics).

[32] Brown I., Mues C. (2012). An experimental comparison of classification algorithms for imbalanced credit scoring data sets. Expert Syst. Appl..

[33] Staal J., Abràmoff M., Niemeijer M., Viergever M., Van Ginneken B. (2004). Ridge-based vessel segmentation in color images of the retina. IEEE Trans. Med. Imaging.

[34] Hsu C.W., Lin C.J. (2002). A comparison of methods for multiclass support vector machines. IEEE Trans. Neural Netw..

[35] Kivinen J., Smola A., Williamson R. (2004). Online learning with kernels. IEEE Trans. Signal Process..

[36] Cantoni V., Galdi C., Nappi M., Porta M., Riccio D. (2015). GANT: Gaze analysis technique for human identification. Pattern Recognit..

